# Preparation of Glutamine-Enriched Fermented Feed from Corn Gluten Meal and Its Functionality Evaluation

**DOI:** 10.3390/foods12234336

**Published:** 2023-12-01

**Authors:** Lei Fan, Xiaolan Liu, Yongping Deng, Xiqun Zheng

**Affiliations:** 1 College of Food Engineering, Harbin University of Commerce, Harbin 150076, China; 2023940923@qqhru.edu.cn; 2 Key Laboratory of Corn Deep Processing Theory and Technology of Heilongjiang Province, College of Food and Bioengineering, Qiqihar University, Qiqihar 161006, China; 913913_monkey@163.com; 3 College of Food, Heilongjiang Bayi Agricultural University, Daqing 163319, China

**Keywords:** by-product, glutamine peptide, intestinal health, gut microbiota

## Abstract

China faces a persistent deficiency in feed protein resources. Enhancing the utilization efficiency of indigenous feed protein resources emerges as a viable strategy to alleviate the current deficit in protein feed supply. Corn gluten meal (CGM), characterized by a high proportion of crude protein and glutamine, is predominantly employed in animal feed. Nonetheless, the water-insolubility of CGM protein hampers its protein bioavailability when utilized as feed material. The aim of this study was to augment protein bioavailability, liberate glutamine peptides from CGM, and produce glutamine-enriched CGM fermented feed. We executed a co-fermentation protocol using *Bacillus subtilis* A5, *Lactobacillus* 02002, and acid protease to generate the CGM fermented feed. Subsequent in vivo experiments with broilers were conducted to assess the efficacy of the fermented product. The findings revealed that the soluble protein, glutamine, small peptides, and lactic acid contents in the fermented feed increased by 69.1%, 700%, 47.6%, and 125.9%, respectively. Incorporating 15% and 30% CGM fermented feed into the diet markedly enhanced the growth performance and intestinal health of broilers, positively modulated the cecal microbiota structure, and augmented the population of beneficial bacteria, specifically *Lactobacillus*. These results furnish both experimental and theoretical foundations for deploying CGM fermented feed as an alternative protein feed resource.

## 1. Introduction

In China, the scarcity of feed protein resources significantly impedes the swift advancement of the feed industry and livestock breeding. Soybean meal (SBM) serves as the primary resource of high-quality protein in animal diets; yet, 80% of the total soybean consumption (precursor of SBM) is dependent on imports. Alternative protein resources, such as cottonseed meal, canola meal, and rapeseed meal, are seldom incorporated into animal diets due to their inferior amino acid profile, higher fiber content relative to SBM, and the presence of macromolecule proteins [1,2,3]. Consequently, enhancing the utilization efficiency of indigenous feed protein resources emerges as a viable strategy to fulfill the existing supply requirements and meet future demands for protein feed resources.

Termed “the second brain”, the small intestine is crucial for nutrient absorption, metabolism, and the recognition of dietary signals. It also establishes a physical and biological defense against detrimental entities, including pathogens and environmental toxins. Disruptions in intestinal architecture and microbiota equilibrium can compromise the intestinal barrier’s functionality, resulting in diminished immunity and stunted growth performance in animals. Glutamine (Gln), a nutrient pivotal for gut health, is integral in promoting intestinal epithelial proliferation, preserving intestinal morphological integrity, and safeguarding the intestinal barrier function [4]. Nonetheless, the direct application of Gln in the feed industry is constrained by its instability and poor water solubility. In contrast, glutamine peptides, known for their enhanced stability and solubility, exhibit superior intestinal modulation capabilities compared to the Gln monomer [5,6] and demonstrate potent immunoregulatory and health-boosting properties as feed additives [7,8,9]. Hence, glutamine peptides represent an efficacious substitute for Gln in protecting intestinal health.

Corn gluten meal (CGM), a significant by-product of the corn starch industry, is primarily utilized in animal feed as a protein source. Over the past five years, China’s annual CGM yield has reached 3 million tons. Comprising approximately 60–70% crude protein, which includes 65% zein and 30% glutelin, the Gln content accounts for about one fourth and one third of the total amino acids in zein and glutelin, respectively [10,11]. Despite its high crude protein content, the bioavailability of CGM protein is restricted due to the water insolubility of zein and glutelin [12]. Moreover, glutamine peptides remain inactive within the CGM protein molecule’s sequence, further limiting the biological functions of Gln in CGM. Solid-state fermentation is an efficient and cost-effective method for altering the physical or physicochemical properties of feedstock, enhancing the bioavailability of agricultural by-products, and generating high-value-added products [13].

Both *Bacillus subtilis* and lactic acid bacteria are extensively employed as fermentation strains. *Bacillus subtilis*, known for its remarkable extracellular protease production, can break down large proteins into smaller proteins and peptides [14]. *Lactobacillus*, part of the lactic acid bacteria family, can reduce the fermentation system’s pH and curb the growth of certain pathogenic bacteria through its organic acid production [15]. The incorporation of exogenous protease during fermentation enriches the protease system, boosting microorganisms’ efficiency in utilizing macromolecular proteins in feed and thereby enhancing protein bioavailability [16]. To augment the bioavailability of CGM protein and liberate its glutamine peptides, this study implemented a co-fermentation protocol using *Bacillus subtilis*, *Lactobacillus*, and acid protease. The bioavailability of the fermented feed was then assessed through an in vivo broiler feeding experiment. Notably, the literature offers no insights into the functionality of glutamine peptides derived from CGM.

## 2. Materials and Methods

### 2.1. Materials

CGM (crude protein, 65%) and corn steep liquor were sourced from Longjiang Fufeng Biotechnologies Co., Ltd. (Qiqihar, China). Wheat bran, SBM, and corn germ meal were obtained from a local market. Acid protease (50,000 U/g; pH 2.0–4.0) was procured from Doing-Higher Bio-Tech Co., Ltd. (Nanning, China). Bis-1,1-trifluoroacetoxy-iodobenzene (BTI) was purchased from Sigma-Aldrich Co. (St. Louis, MO, USA). MRS (de Man, Rogosa, and Sharpe) agar and LB (Luria-Bertani) agar were purchased from Hope Bio-Technology Co., Ltd. (Qingdao, China). Skimmed-milk agar was prepared as follows: 25 g of nonfat dry milk (High-Protein Skim Milk Powder, Yili Industrial Group Limited by Share Ltd., Beijing, China) was reconstituted with 250 mL of distilled water. The mixture was stirred thoroughly and autoclaved at 115 °C for 15 min. Similarly, 250 mL of 4% agar solution was autoclaved at 121 °C for 15 min. The skimmed milk was then combined with the agar solution and thoroughly mixed at 50–60 °C, before being quickly poured into plates. CGM screening agar was composed of 3% CGM and 2% agar. MRS-CaCO_3_ plates were prepared using MRS agar containing 2% CaCO_3_.

**Figure A1:**
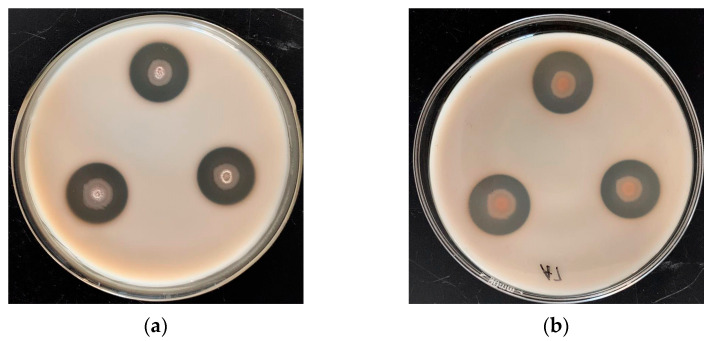

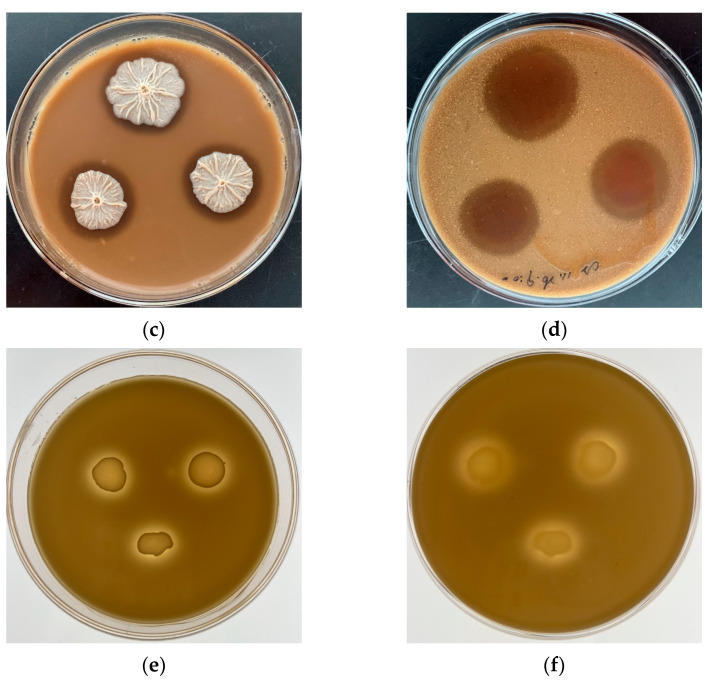
CGM degradation and acid production capabilities of *Bacillus subtilis* A5 and *Lactobacillus* 02002. The hydrolysis circle of *Bacillus subtilis* A5 on the front (**a**) and back (**b**) of skimmed-milk agar plates; the hydrolysis circle of *Bacillus subtilis* A5 on the front (**c**) and back (**d**) of corn gluten meal plates; the hydrolysis circle of *Lactobacillus* 02002 on the front (**e**) and back (**f**) of MRS-CaCO_3_ plates.

*Bacillus subtilis* A5 and *Lactobacillus* 02002 were selected based on their CGM degradation and acid production capabilities. Briefly, for *Bacillus subtilis*, strains were applied to the surface of 10% (*w*/*v*) skimmed-milk agar using a sterility toothpick and incubated at 37 °C for 48 h to select strains with a larger ratio of transparent diameter to colony diameter. These strains were then placed on the surface of a CGM screening plate to identify the strain with the largest ratio of transparent circle diameter to colony diameter. For *Lactobacillus,* 5 µL of lactic acid bacteria broth (incubated for 16 h) was applied to the surface of MRS-CaCO_3_ agar and incubated for 24 h to select the strain with the largest transparent circle diameter. Appendix A Figure A1 shows the CGM degradation and acid production capabilities of *Bacillus subtilis* A5 and *Lactobacillus* 02002. All strains were preserved at the laboratory of the Department of Food and Biological Engineering at the University of Qiqihar (Qiqihar, China).

### 2.2. Glutamine-Enriched CGM Fermented Feed Preparation

Initially, a blend was created using 40% CGM (extruded and milled), 10% SBM, 10% wheat bran, and 40% corn germ meal. Subsequently, a combined bacterial solution was prepared using *Bacillus subtilis* A5 (2 × 10^8^ CFU/mL) and *Lactobacillus* 02002 (2 × 10^7^ CFU/mL) in a 1:1 volume ratio. Sterile water, the mixed bacterial solution, acid protease liquid (1000 U/mL), and corn steep liquor were then added to the blend to produce a final fermentation mixture with 45% moisture content (*v*/*w*), a 3% bacterial inoculation concentration (*v*/*w*), 50 U/g acid protease amount, and 10% corn steep liquor (*v*/*w*). Finally, the fermentation mixture was packaged in multi-layer, polythene bags equipped with a one-way valve and incubated at 30 °C for 72 h to obtain the glutamine-enriched CGM fermented feed (GCFF).

### 2.3. Nutritional Value Evaluation of the Glutamine-Enriched CGM Fermented Feed

The feed samples were dried at 65 °C for 48 h and ground using an extruder. The dry matter (method 930.15), crude protein (method 976.05), and crude fat (method 920.39) contents were subsequently analyzed, as described by the Association of Official Analytical Chemists (AOAC 1990) [17]. The crude fiber, acid detergent fiber, and neutral detergent fiber contents were determined according to the standard of GB/T 6434-2022 [18], NY/T 1459-2022 [19], and GB/T 20806-2022 [20], respectively. The pH value was assessed based on standard GB/T 10468-1989 [21]. Soluble protein content was evaluated using the Folin-phenol method, while small peptides were quantified via the trichloroacetic acid method outlined by Wang et al. [22]. Lactic acid content was measured using an ion chromatograph (Dionex ICS-1100, Thermo Fisher, Waltham, MA, USA). Probiotic populations were assessed using the plate-counting method. Briefly, feed sample was serially diluted (1:10) to 10^−7^, and these dilutions were plated in triplicate onto LB agar for *Bacillus subtilis* and onto MRS agar for *Lactobacillus*. CFUs on the agars were counted and are expressed as CFU per gram of wet feed.

### 2.4. Glutamine Content Evaluation

The Gln content in soluble protein was determined using the method described in our previous report [11]. The glutamine peptide content was characterized by assessing the *no-N* terminal Gln content, as *N* terminal Gln in an aqueous solution easily converts to pyroglutamic acid, losing the beneficial effects of Gln. Briefly, 50 g of dried and ground feed was added to 500 mL of solvent, and was then allowed to stand at room temperature for 2 h before being centrifuged at 10,000 r/min for 30 min. The supernatant was filtered using filter paper and then freeze-dried to obtain the feed extract powder. Subsequently, 500 μL of feed extract (100 mg/mL), 2 mL BTI acetonitrile-aqueous solution (10 mg/mL), and 500 μL of pyridine (50 μmol/mL) were combined in an ampoule to create a reaction mixture. The Gln protect reaction of mixture was carried out at 50 °C for 2 h, and a control mixture was prepared without the BTI protection reaction. The ampoules were then vacuum-sealed and subjected to an acid-hydrolyzed reaction (110 °C for 24 h) following the addition of 5 mL HCl (6 mol/L). After cooling, the glutamic acid content was measured using a glutamate biosensor analyzer (M-100, Sieman Technology Co., Ltd., Shenzhen, China), with the pH adjusted to neutral. The glutamine content was inferred from the difference in glutamic acid levels between the two samples.

### 2.5. Animal, Design, and Diets

The animal experiments were approved on 20 June 2022 by the Animal Ethics Committee of the College of Food and Bioengineering, Qiqihar University (Qiqihar, China) (Approval No. 2022-012). A total of 160 one-day-old Arbor Acre broilers were acquired from Qiqihar Deyu Animal Husbandry Co., Ltd. (Qiqihar, China), with an initial body weight (BW) of 41.80 ± 0.23 g. The broilers were randomly divided into four groups, each with five replicates of eight birds. The four experimental treatments were as follows: (1) corn-soybean meal basal diet (CON), (2) diet with 15% of SBM replaced by GCFF (GCFF15), (3) diet with 30% of SBM replaced by GCFF (GCFF30), (4) diet with 60% of SBM replaced by GCFF (GCFF60). The diets were designed to contain similar levels of metabolizable energy and crude protein, adhering to the National Research Council (1994) guidelines to meet the nutritional needs of the animals. The ingredient and chemical composition of the basal diets are presented in Table 1. The feeding program comprised two phases: days 1 to 21 (starter phase) and days 22 to 42 (finisher phase). Room temperature was maintained at 33 ± 1 °C for the first 7 days, and was then gradually decreased to 22 ± 1 °C by the end of the trial. All broilers had ad libitum access to feed and water throughout the 42-day experimental period.

### 2.6. Sample Collection

On days 21 and 42, broilers were fasted for 12 h prior to sampling. Broilers were weighted by replication, and feed consumption was recorded similarly. The BW of the broilers was recorded to calculate the average daily gain (ADG), and feed consumption was monitored to determine the average daily feed intake (ADFI). Subsequently, the feed conversion rate (FCR) was calculated. On day 42, two broilers of average BW were randomly selected from each replicate, stunned, and exsanguinated from the jugular vein, with samples of intravenous blood being collected. The blood samples were then centrifuged at 3000 g for 15 min at 4 °C, and the serum was harvested and stored at −20 °C for serum parameter analysis. The small intestines were removed, and approximately 2–3 cm segments from the middle part of the jejunum and ileum were excised. The intestinal segments were rinsed with ice-cold saline water and divided into two parts. One part was fixed in 4% paraformaldehyde solution for intestinal morphology measurement, and the other part was immediately frozen in liquid nitrogen and stored at −80 °C for measurement of the tissues’ immune parameters. The lengths and weights of the duodenum, jejunum, and ileum segments were measured. Cecal contents were aseptically collected, placed in sterilized tubes, immediately frozen in liquid nitrogen, and stored at −80 °C for 16S rRNA gene amplicon deep sequencing.

### 2.7. Intestinal Morphometry Analysis

The small intestinal samples were fixed in 4% paraformaldehyde solution for 24 h. Tissues were embedded in paraffin wax blocks, mounted onto glass slides, and stained with hematoxylin and eosin. The images of the small intestines were acquired using an Olympus microscope (Olympus Optical Co., Ltd., Beijing, China). Villus height (VH) and crypt depth (CD) were quantified using Image-Pro Plus software 6.0 (Media Cybernetics, Inc., Washington, DC, USA). The VH-to-CD ratio (VCR) was calculated. VH was measured from the top of the villus to the villus–crypt junction, and CD was measured from the base of the crypt up to the crypt junction. Morphometric measurements were performed on 6 intact villi and corresponding crypts in each slice. The relative weight and length of the small intestine were calculated using the following formulas, respectively:

Relative intestine weight (g/100 g BW) = (intestine weight × 100)/broiler weight.

Relative intestine length (cm/100 g BW) = (intestine length × 100)/broiler weight.

### 2.8. Determination of Serum and Jejunum Tissues’ Immunological Parameters

Levels of IgG, IgA, and IgM in serum, jejunum tissues, and ileum tissues were quantified using chicken-specific ELISA kits (Jianglai Biotechnology Co., Ltd., Shanghai, China), adhering to the manufacturer’s protocols.

### 2.9. S rRNA High Throughput Sequencing

Total genomic DNA was extracted from cecal samples utilizing a DNA isolation kit (Jianglai Biotechnology Co., Ltd., Shanghai, China). Alterations in intestinal microbiota composition were examined through 16S rRNA sequencing analysis. Data were processed using the Majorbio Cloud Platform (Majorbio, Shanghai, China).

### 2.10. Statistical Analysis

All data were analyzed using GraphPad Prism 8.0.2 (Graph Pad Software Inc., San Diego, CA, USA). The chemical compositions of unfermented and fermented feeds were compared using the unpaired *t*-test procedure. The animal experimental data were evaluated using a one-way ANOVA procedure. Tukey’s multiple range test was used to identify significant differences between treatment variables. Differences were considered statistically significant at *p* < 0.05. Results are presented as mean ± standard error of the mean (SEM). All experiments were conducted at least three times.

## 3. Results

### 3.1. Chemical Compositions of the GCFF

Table 2 presents the chemical composition of the GCFF. Post fermentation, the contents of soluble protein and small peptides were increased by 69.1% and 47.6%, respectively, while the levels of crude fiber, crude fat, and acid detergent fiber significantly diminished (*p* < 0.05). The pH value decreased from 5.2 to 4.3, accompanied by a notable increase in lactic acid content by 125.9% (*p* < 0.05). Populations of *Lactobacillus* and *Bacillus subtilis* reached 2.6 × 10^6^ and 1.1 × 10^5^, respectively. The rise in soluble protein and small peptides resulted from the combined degradation activity of microbial protease and acid protease on macromolecule proteins. These low-molecular-weight proteins and peptides are more readily absorbed and digested by animals. The reduction in crude fiber, crude fat, and acid detergent fiber can be attributed to the degradation action of cellulase and lipase, produced by *Bacillus subtilis* A5. The decline in pH value was primarily due to the lactic acid generated by *Lactobacillus* 02002, which may also impart a distinctive sour aroma to the feed.

### 3.2. Glutamine Content of the GCFF

The BTI protection method was employed to ascertain the Gln content in the soluble protein. As depicted in Table 2, the Gln content within the soluble protein of the GCFF significantly rose from 1.6 mg/g to 12.8 mg/g (*p* < 0.05), evidencing the successful liberation of glutamine peptides from CGM protein.

### 3.3. Effects of Dietary GCFF Replacing SBM Supplementation on the Growth Performance of Broilers

Table 3 outlines the effects of GCFF substitution for SBM on broilers’ growth performance. Broilers fed diets with 15% and 30% GCFF replacing SBM showed a higher ADG compared to the basal group throughout the entire period. There was no significant difference in ADFI values for broilers fed diets with 15% and 30% GCFF replacements compared to those on the CON diet during the entire period. FCR values for broilers on the 15% and 30% GCFF diets were lower than those on the CON diet throughout the period. However, broilers fed a diet with 60% GCFF replacing SBM exhibited a lower ADG and ADFI and higher FCR compared to the basal group during the entire period. These findings suggest that 15% and 30% GCFF substitutions for SBM may enhance growth performance, while a 60% substitution may adversely affect performance.

### 3.4. Effects of Dietary GCFF Replacing SBM Supplementation on the Intestinal Morphometry of Broilers

**Figure 1:**
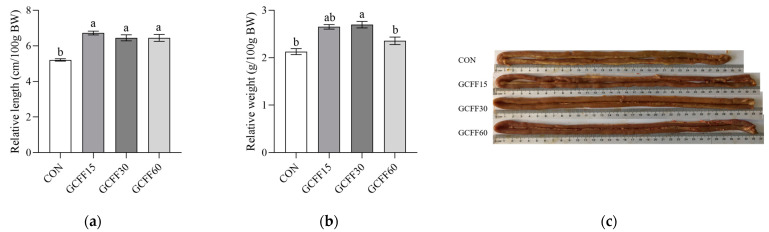

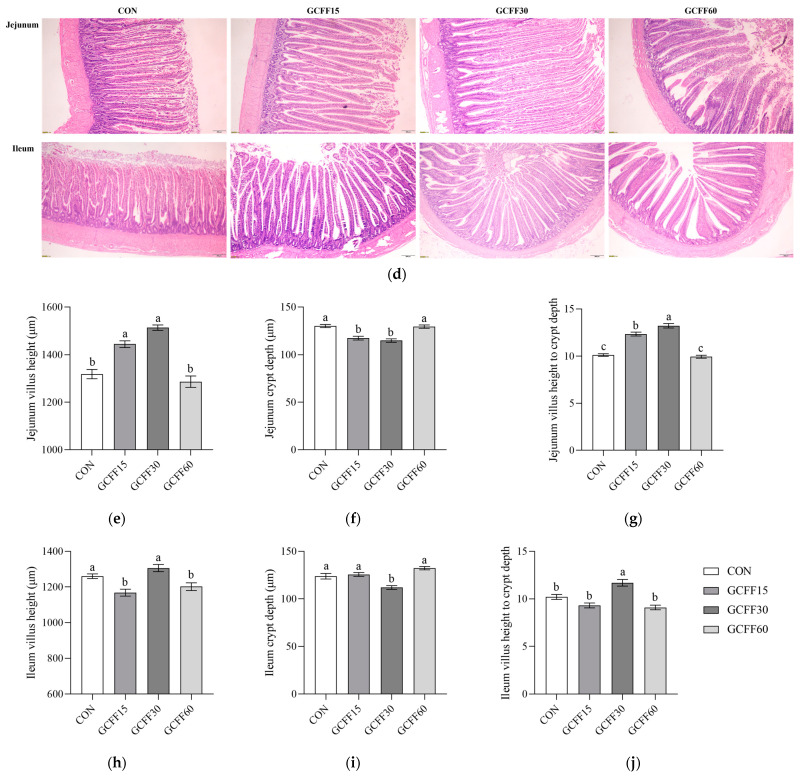
Intestinal morphometry of broilers fed experimental diets with or without GCFF replacing SBM. (**a**) Relative length of small intestines; (**b**) relative weight of small intestines; (**c**) jejunum length on day 42; (**d**) morphology of jejunum and ileum of broilers (4 × magnification); (**e**) jejunum villus height; (**f**) jejunum crypt depth; (**g**) jejunum villus height to crypt depth; (**h**) ileum villus height; (**i**) ileum crypt depth; (**j**) ileum villus height to crypt depth. CON, corn and soybean meal basal diet; GCFF, glutamine-enriched corn gluten meal fermented feed; GCFF15, diet with 15% of soybean meal substituted with GCFF; GCFF30, diet with 30% of soybean meal substituted with GCFF; GCFF60, diet with 60% of soybean meal substituted with GCFF. a–c, values with the same letter mean no significant difference between groups (*p* > 0.05), while values with different letters mean significant difference (*p* < 0.05) between groups.

The integrity of the intestinal physical barrier is pivotal for animal health. Essential markers of intestinal structural integrity, such as intestinal length, VH, CD, and VCR, directly influence the absorptive efficiency of the intestinal mucosa. Figure 1 illustrates the effect of GCFF substitution for SBM on the morphological structure of broilers’ small intestines. Diets with GCFF substituting for SBM increased the relative length and relative weight of the small intestine (Figure 1a–c). The histometric measurements of VH and CD in the jejunum and ileum of broilers are shown in Figure 1d. In the jejunum, broilers on the 15% and 30% GCFF diets exhibited a significantly higher VH and lower CD compared to those on the CON diet, with the 30% GCFF group displaying the highest VCR among all cohorts (Figure 1e–g). Regarding the ileum, the GCFF30 cohort demonstrated the highest VH and VCR and the lowest CD (Figure 1h–j). These findings suggest that appropriate GCFF substitution for SBM can promote intestinal structural maturation and enhance the physiological health of the intestine.

### 3.5. Effects of Dietary GCFF Replacing SBM Supplementation on the Serum, Jejunum, and Ileum Tissues’ Immunoglobulins of Broilers

Serving as the initial defense barrier against pathogenic intrusion, immunoglobulins are integral to the body’s immune competence. Table 4 reveals the effects of GCFF substitution for SBM on serum, jejunum and ileum tissues’ immunoglobulins. Dietary substitution with GCFF led to a rise in serum IgG levels, though no significant alterations were observed in IgM and IgA concentrations. In the jejunal tissues, levels of IgG, IgM, and IgA significantly escalated following GCFF substitution for SBM (*p* < 0.05). In the ileum tissues, the levels of IgG and IgM in broilers fed with GCFF were significantly higher than those in broilers fed the CON diet, while the IgA levels showed no significant difference. These results denote an enhancement in the broilers’ humoral immunity attributable to the GCFF substitution for SBM.

### 3.6. Effects of Dietary GCFF Replacing SBM Supplementation on the Intestinal Microbiota of Broilers

**Figure 2:**
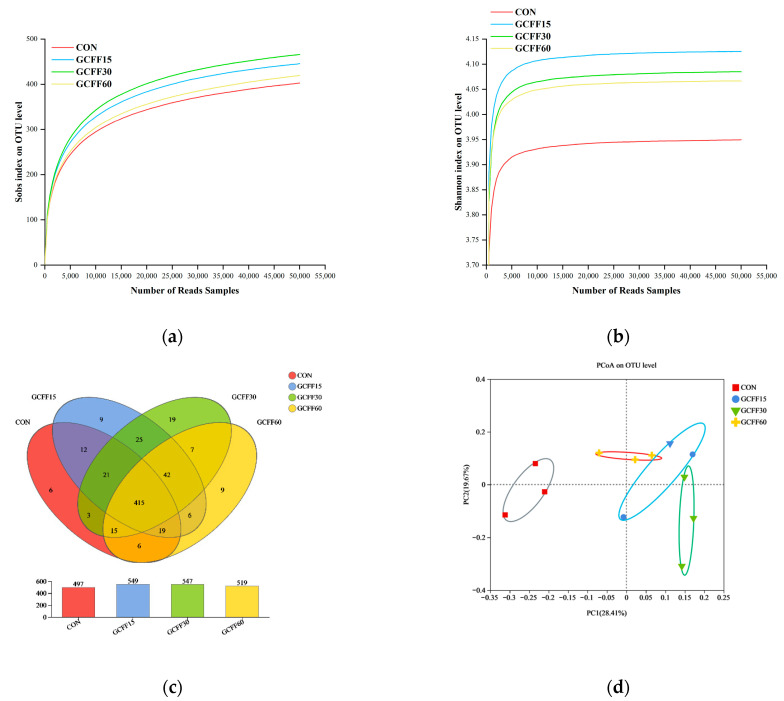

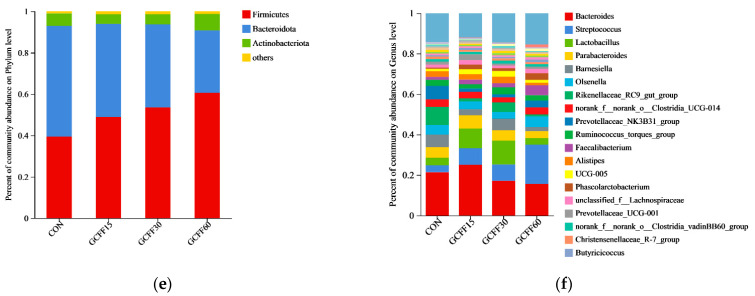
Intestinal microbiota of broilers fed experimental diets with or without GCFF replacing SBM. (**a**) Sobscurves; (**b**) Shannon curves; (**c**) Venn diagrams of OTU; (**d**) principal coordinate analysis (PCoA); (**e**) microbiota analysis at the phylum level; (**f**) microbiota analysis at the genus level. CON, corn and soybean meal basal diet. GCFF, glutamine-enriched corn gluten meal fermented feed; GCFF15, diet with 15% of soybean meal substituted with GCFF; GCFF30, diet with 30% of soybean meal substituted with GCFF; GCFF60, diet with 60% of soybean meal substituted with GCFF.

The gut microflora consists of billions of microorganisms, forming a mutually dependence and restriction dynamic balance within the intestinal micro-ecosystem. Healthy intestinal microflora benefits the host by modulating nutrient absorption and maintaining intestinal microecological stability. The cecal bacterial community in broilers was analyzed using 16S rRNA high-throughput sequencing, with results presented in Figure 2. The Sobs and Shannon index serve as indicators of alpha-diversity, describing microbial diversity within a specific region or ecosystem. A Venn diagram was constructed to identify specific and shared operational taxonomic units (OTUs) in broilers’ cecal samples under various diets. Principal coordinate analysis (PCoA) was employed to discern similarities or differences between groups. This study yielded a total of 723,969 effective sequences post-quality control filtration, clustering into 614 OTUs at 97% sequencing identity. Both the Sobs index (Figure 2a) and Shannon index (Figure 2b) were higher in the groups with GCFF replacing SBM compared to the CON group, suggesting that dietary GCFF supplementation in place of SBM could enhance the complexity of broilers’ intestinal microflora. The groups with GCFF replacing SBM harbored more OTUs than the CON group (Figure 2c), indicating that GCFF supplementation in lieu of SBM could enrich the diversity of intestinal microbiota in broilers. Regarding the PCoA plot, the groups with GCFF replacing SBM clustered closely together, separated distinctly and substantially from the CON group (Figure 2d), demonstrating that dietary GCFF replacing SBM supplementation altered the microbial composition.

Intestinal microbiota abundance at the phylum level is depicted in Figure 2e. *Firmicutes*, *Bacteroidota,* and *Actinobacteria* comprised over 98% of the four phyla in each treatment group. The relative abundance of *Bacteroidota* was greater in the CON group compared to the groups with GCFF replacing SBM, while *Firmicutes* were significantly less abundant in the CON group. At the genus level (Figure 2f), GCFF markedly affected broilers’ intestinal microflora. The relative abundance of the *Lactobacillus*, *Streptococcus*, and *Parabacteroides* were significantly higher in the groups with GCFF replacing 15% and 30% of SBM than in the CON group.

## 4. Discussion

Previous studies have affirmed that supplementation with Gln or glutamine peptides can enhance intestinal morphology, immune responses, intestinal microbiota, and, subsequently, the growth performance of animals. Appendix A Table A1 shows partial corn protein sequence information in UniProt. Recognized as a superior source of glutamine-enriched protein, CGM undergoes extrusion, milling, and subsequent co-fermentation with *Bacillus subtilis* A5, *Lactobacillus* 02002, and acid protease to produce glutamine-enriched fermented feed. Our findings indicate that the co-fermentation process markedly elevates the concentration of glutamine peptides in the substrate. Firstly, during CGM pre-treatment, the extrusion process disrupts the protein bodies within CGM and unfolds the protein structures, enhancing protease accessibility for protein hydrolysis. These outcomes align with those of Zheng et al., who observed that Alcalase more readily hydrolyzed extruded CGM compared to its non-extruded counterpart [23]. Secondly, *Bacillus subtilis* A5, chosen for its potent CGM protein degradation capability, facilitates the conversion of parent proteins into active peptides, including glutamine peptides. It has also been reported that *Bacillus pumilus* secretes alkaline protease, demonstrating a robust capacity to hydrolyze zein [24]. Thirdly, acid protease pre-digests CGM protein and disrupts interactions between proteins and other constituents, thereby enhancing the efficacy of microbial protease [25]. Wang et al. reported that rapeseed meal fermented with the assistance of acid protease contained a higher peptide concentration than that fermented solely by microorganisms [3]. Finally, the acidic environment created by organic acids from *Lactobacillus* 02002 optimizes acid protease catalytic activity, leading to an increase in CGM protein degradation efficiency [26]. The augmented presence of glutamine peptides and other small peptides due to fermentation is likely to benefit both gut health and production performance in broilers.

The results of the current study reveal that the dietary supplementation of GCFF, replacing 15% and 30% of SBM, enhanced the ADG and ADFI values in broilers. These findings align with Zhou’s previous work, which noted an improved daily weight gain in early-weaned calves receiving a diet supplemented with 1.01 g/kg of alanyl-glutamine [27]. Similarity, Abdulkarimi et al. found that 0.5% Gln supplementation boosted both ADG and ADFI in broilers [28], while Wang et al. reported that a diet containing 10% fermented CGM increased the ADG in three yellow broilers [1]. Moreover, this study observed a reduction in FCR values when GCFF replaced 15% and 30% of SBM in the diet, suggesting that this substitution could reduce costs and enhance economic benefits. The augmented growth performance in broilers due to the inclusion of fermented diets may be attributable to the heightened nutritional bioavailability within the fermented feed, including a greater proportion of soluble protein and small peptides. These components are readily absorbed, digested, and ultimately deposited into muscle tissue. Additionally, the lactic acid imparted an acidic flavor to the feed, improving its palatability, which in turn likely increased broilers’ feed intake and accelerated the rate of nutrient deposition, thereby promoting body weight gain. To elucidate the mechanisms underlying the growth-promoting effects of GCFF, this study further probed the intestinal responses of broilers to GCFF diets.

A robust intestinal structure is pivotal for the growth promotion in animals. An elongated intestinal length, increased intestinal weight, elevated VH, and reduced CD suggest enhanced nutrient digestion and absorption within the small intestine. This research determined that diets with GCFF substituting for SBM augmented intestinal length and VH, diminished CD, and consequently, raised the VCR ratio (Figure 1). These outcomes are consistent with our findings; Gln supplementation improved the relative weight and length of the intestine, as well as the VH and VCR of the Nile tilapia juveniles [29]. Jing et al. reported that a CGM hydrolysate rich in glutamine peptides mitigated colonic shortening and decreased the permeability of the colonic mucosa in mice [11]. The beneficial effects of GCFF replacing SBM diets on gut morphology are presumably linked to the metabolic activities of Gln present in the fermented feed. Gln can supply energy and metabolic precursors for the proliferation and differentiation of intestinal mucosal cells, facilitate mucosal protein synthesis, regulate intestinal tight junction permeability, and thereby enhance intestinal structure and function comprehensively [30]. Indeed, glutamine peptides have been shown to more effectively stimulate enterocyte proliferation than the Gln monomer.

In harmony with the positive impact of GCFF on intestinal morphology, an enhanced immunological function was noted in the serum, jejunum tissues, and ileum tissues of broilers supplemented with GCFF replacing SBM. The bolstering of the immune system plays a crucial role in maintaining intestinal mucosal homeostasis and the host’s physiological functions [31]. Gln has been documented to safeguard intestinal health and augment growth performance by modulating the immune response [32,33]. Corroborating our findings, Zhou et al. discovered that intravenously administered glutamine peptides elevated serum IgA, IgG, and intestinal mucosal sIgA, culminating in an advantageous impact on weight gain and intestinal structural integrity in early-weaned calves [27]. Similarly, Xu et al. found that dietary supplementation with glutamine peptides heightened the serum concentrations of IgA, IgG, and IgM, thereby more effectively enhancing intestinal barrier function [9]. The underlying mechanism bolstering immunity may be attributed to the Gln or glutamine peptides present in the fermented feed. Gln, recognized as a potent immune stimulant, serves as an essential energy source for innate immune cells, including lymphocytes and macrophages. The proliferation and activation of these immune cells lead to the secretion of various antibodies, thereby regulating the immune response and fostering a robust immune system.

Given the gut microbiota’s pivotal role in sustaining intestinal health, orchestrating immune responses, and promoting growth, it is imperative to elucidate the effects of GCFF on broilers’ microbiota composition. Currently, a greater diversity and richness in gut bacteria are considered advantageous, helping animals navigate various environmental challenges. Gln and glutamine peptides have been reported to preserve intestinal structure and function, in addition to enhancing growth performance, by modulating gut microbiota [34]. In this study, microbial diversity, richness, and community abundance experienced a significant upsurge when GCFF replaced 15% and 30% of SBM (Figure 2). This observation is consistent with findings that dietary glycyl-glutamine supplementation augmented the alpha-diversity of gut microbiota, thereby improving growth performance indices [35]. Comparable outcomes were documented with alanyl-glutamine peptide and Gln monomer supplementation [9]. Jing et al. also determined that CGM hydrolysate rich in glutamine peptides could regulate the intestinal microbiota’s abundance and diversity, mitigate weight loss, and lower colonic mucosa permeability in mice [11]. Gln orchestrates the survival and proliferation of intestinal bacteria through the modulation of amino acid bacterial metabolism [36]. Known as a precursor in arginine biosynthesis, Gln has been shown to influence the metabolism of arginine family amino acids in the small intestine, potentially mediated in part by small-intestinal bacteria [37]. It is hypothesized that glutamine peptides might alter the bacterial utilization and metabolism of arginine-family amino acids within the intestine, thereby affecting bacterial activity and population dynamics, and prompting a shift in the community of commensal bacteria toward those more beneficial for host metabolism. Besides bioactive peptides, probiotics such as *Lactobacillus* and *Bacillus subtilis* might also contribute to the observed changes in broilers’ intestinal microbial populations. These probiotics can thwart the colonization of harmful pathogens and promote the proliferation of beneficial anaerobic bacteria through the secretion of short-chain fatty acids and bacteriocins, ultimately enhancing mucosal morphology and the overall health of the animals [38,39]. In summary, these findings suggest that GCFF supplementation can positively alter intestinal microflora structure, although further investigation is warranted to ascertain the precise mechanism.

Gut commensal microbes are integral to maintaining the physiologic and metabolic homeostasis of the host, particularly in preserving intestinal health, owing to their efficiency in nutrient absorption and utilization [40]. In this study, at the phylum level, the *Firmicutes*-to-*Bacteroidetes* ratio escalated in tandem with an increasing proportion of GCFF replacing SBM (Figure 2). A higher *Firmicutes*-to-*Bacteroidets* ratio correlates with an augmented energy harvest and enhanced growth performance. Prior research indicated that a diet deficient in protein and energy led to a reduction in *Firmicutes* and a surge in *Bacteroidets* [41]. Given the results of the current study, it can be inferred that following the introduction of fermented feed, modifications in intestinal microflora enhanced the efficiency of nutrient absorption and utilization, including proteins and peptides. At the genus level, populations of *Lactobacillus*, *Streptococcus*, and *Parabacteroides* expanded in correlation with the proportion of GCFF supplanting SBM. *Lactobacillus* possesses a robust ability to metabolize carbohydrates into organic acids, thereby inhibiting pathogen growth, modulating gut immune function, and promoting host health [41]. Bacterial species, including *Lactobacillus*, *Streptococcus.* and *Parabacteroides,* exhibit proficiency in protein degradation [42,43]. *Streptococcus*, a bacterium involved in amino acid fermentation, utilizes Gln as its preferred nitrogen source [44] and exhibits high activity in dipeptidyl dipeptidase [45]. Our data suggest that an optimal ratio of GCFF replacing SBM in diets could significantly bolster the population of proteolytic bacteria, enhancing nutrient uptake efficiency, and ultimately improving the intestinal structure integrity and growth performance of broilers.

## 5. Conclusions

In conclusion, the results of this study clearly demonstrate that co-fermentation enhances the soluble protein, small peptides, and glutamine peptide release in CGM, establishing CGM as an optimal raw material for glutamine peptide preparation. Dietary supplementation with 15% and 30% GCFF in lieu of SBM can improve growth performance, boost immune function, positively affect gut microbial flora structure, and enhance intestinal health in broilers. GCFF emerges as a promising alternative protein source in feed. However, this study did not elucidate the specific mechanisms promoting broiler growth, and subsequent research will aim to uncover the health-promoting mechanisms of glutamine peptides. This research presents an innovative approach for creating a functional feed additive with growth-promoting properties and proposes a viable solution to mitigate the scarcity of feed protein resources.

## Figures and Tables

**Table 1:** Composition of experimental diets.

Item	1–21 Days				22–42 Days			
	CON	GCFF15	GCFF30	GCFF60	CON	GCFF15	GCFF30	GCFF60
Ingredients (%, as-fed basis)								
Corn	57.49	59.77	57.47	57.97	62.00	63.40	62.00	62.40
Soybean meal	35.80	29.63	25.06	14.32	31.07	26.41	21.75	12.43
CGM fermented feed	0.00	5.37	10.74	21.48	0.00	4.66	9.32	18.65
Soybean oil	3.00	2.52	3.00	2.32	3.60	2.20	3.60	3.00
Limestone	1.27	1.27	1.27	1.27	1.21	1.21	1.21	1.21
Dicalcium phosphate	1.45	1.45	1.45	1.45	1.20	1.20	1.20	1.20
Choline chloride	0.20	0.20	0.20	0.20	0.20	0.20	0.20	0.20
NaCl	0.35	0.35	0.35	0.35	0.37	0.37	0.37	0.37
Premix ^1^	0.22	0.22	0.22	0.22	0.22	0.22	0.22	0.22
DL-Methionine (98%)	0.20	0.20	0.20	0.20	0.12	0.12	0.12	0.12
L-Lysine-HCl (99%)	0.02	0.02	0.04	0.20	0.01	0.01	0.01	0.20
Nutrient levels (Calculate values)								
Metabolizable energy (MJ/kg)	12.37	12.38	12.54	12.53	12.72	12.70	12.86	12.85
Crude protein (%)	20.42	20.17	20.42	20.46	18.69	18.72	18.69	18.73
Crude fat (%)	5.69	5.34	5.87	5.40	6.35	6.05	6.50	6.08
Crude fiber (%)	3.43	3.33	3.26	3.10	3.26	3.19	3.11	2.96
Total phosphorus (%)	0.80	0.80	0.79	0.79	0.71	0.71	0.70	0.70
Calcium (%)	1.90	1.91	1.87	1.86	1.89	1.89	1.87	1.85
Lysin (%)	1.12	1.01	0.96	0.96	0.99	0.91	0.83	0.87
Methionine (%)	0.50	0.51	0.53	0.55	0.41	0.42	0.43	0.45

Note: ^1^ The premix provided the following per kilogram of diet: Vitamin A, 9500U; Vitamin D3, 0.0625 mg; Vitamin K3, 2.65 mg; Vitamin B1, 0.025 mg; Vitamin B2, 6 mg; Vitamin E, 30 U; biotin, 0.0325 mg; folic acid, 1.25 mg; pantothenic acid, 12 mg; niacin, 50 mg; Cu, 8 mg; Zn, 75 mg; Fe, 80 mg; Mn, 100 mg; Se, 0.15 mg; I, 0.35 mg. CON, corn and soybean meal basal diet; GCFF, glutamine-enriched corn gluten meal fermented feed; GCFF15, diet with 15% of soybean meal substituted with GCFF; GCFF30, diet with 30% of soybean meal substituted with GCFF; GCFF60, diet with 60% of soybean meal substituted with GCFF.

**Table 2:** The chemical analysis of the UFF and GCFF.

Item	UFF	GCFF
Dry matter (%)	90.52 ± 0.19	90.13 ± 0.28
Crude protein (%)	40.46 ± 0.28	41.33 ± 0.46
Crude fiber (%)	12.98 ± 0.09 ^a^	9.10 ± 0.04 ^b^
Crude fat (%)	2.50 ± 0.11 ^a^	2.17 ± 0.04 ^b^
Acid detergent fiber (%)	28.36 ± 0.18 ^a^	23.53 ± 0.25 ^b^
Neutral detergent fiber (%)	8.43 ± 0.12	8.22 ± 0.09
pH	5.2 ^a^	4.6 ^b^
Glutamine content (mg/g soluble protein)	1.60 ± 0.16 ^b^	12.80 ± 0.14 ^a^
Small peptide content (mg/g)	76.10 ± 0.96 ^b^	112.30 ± 1.05 ^a^
Soluble protein content (mg/g)	82.40 ± 0.82 ^b^	140.70 ± 1.98 ^a^
Lactic acid content (mg/g)	13.18 ± 0.03 ^b^	29.78 ± 0.19 ^a^
*Lactobacillus* (CFU/g)	Undetected	2.6 × 10^6^
*Bacillus subtilis* (CFU/g)	Undetected	1.1 × 10^5^

Note: UFF, corn gluten meal unfermented feed; GCFF, glutamine-enriched corn gluten meal fermented feed. ^a^, ^b^, different lowercase superscripts indicate significant differences (*p* < 0.05) in the same row.

**Table 3:** Growth performance of broilers fed experimental diets with or without GCFF replacing SBM.

Item	Time	CON	GCFF15	GCFF30	GCFF60	SEM	*p*-Value
ADG (g)	1–21 days	34.0 ^c^	37.4 ^a^	35.8 ^b^	33.4 ^c^	0.919	<0.001
	22–42 days	82.9 ^c^	92.0 ^a^	88.3 ^b^	76.0 ^d^	3.491	<0.001
	1–42 days	60.7 ^c^	66.3 ^a^	62.2 ^b^	54.6 ^d^	2.413	<0.001
ADFI (g)	1–21 days	53.3	54.5	54.9	52.3	0.599	0.063
	22–42 days	172.7 ^a^	173.2 ^a^	173.5 ^a^	160.2 ^b^	3.237	<0.001
	1–42 days	113.0 ^a^	113.8 ^a^	114.2 ^a^	106.2 ^b^	1.877	<0.001
FCR	1–21 days	1.56	1.52	1.52	1.60	0.019	0.156
	22–42 days	1.98 ^b^	1.82 ^c^	1.98 ^b^	2.08 ^a^	0.054	<0.001
	1–42 days	1.86 ^b^	1.74 ^c^	1.84 ^b^	1.94 ^a^	0.042	<0.001

Note: CON, corn and soybean meal basal diet; GCFF, glutamine-enriched corn gluten meal fermented feed; GCFF15, diet with 15% of soybean meal substituted with GCFF; GCFF30, diet with 30% of soybean meal substituted with GCFF; GCFF60, diet with 60% of soybean meal substituted with GCFF. ADG, average daily gain; ADFI, average daily feed intake; FCR, feed conversion rate. ^a–d^, different lowercase superscripts indicate significant differences (*p* < 0.05) in the same row.

**Table 4:** Immunological parameters of broilers fed experimental diets with or without GCFF replacing SBM.

Item	CON	GCFF15	GCFF30	GCFF60	SEM	*p*-Value
Serum						
IgM (μg/mL)	624.9	632.2	624.9	605.1	5.039	0.414
IgG (μg/mL)	1793.9 ^b^	2135.9 ^a^	2039.5 ^a^	2056.6 ^a^	63.997	0.006
IgA (μg/mL)	215.0	232.9	207.4	205.8	5.376	0.096
Jejunum tissues						
IgM (μg/mL)	548.6 ^b^	603.8 ^a^	605.9 ^a^	566.2 ^b^	12.269	<0.001
IgG (μg/mL)	1609.9 ^b^	1793.5 ^a^	1850.6 ^a^	1798.3 ^a^	45.617	<0.001
IgA (μg/mL)	210.4 ^b^	238.0 ^a^	226.9 ^a^	220.9 ^ab^	4.994	0.005
Ileum tissues						
IgM (μg/mL)	580.4 ^b^	636.0 ^a^	628.9 ^a^	580.8 ^b^	15.038	0.003
IgG (μg/mL)	1383.7 ^b^	1635.4 ^a^	1723.2 ^a^	1629.0 ^a^	72.948	0.001
IgA (μg/mL)	224.1	230.1	238.1	213.7	5.140	0.108

Note: CON, corn and soybean meal basal diet; GCFF, glutamine-enriched corn gluten meal fermented feed; GCFF15, diet with 15% of soybean meal substituted with GCFF; GCFF30, diet with 30% of soybean meal substituted with GCFF; GCFF60, diet with 60% of soybean meal substituted with GCFF. IgM, immunoglobulin M; IgG, immunoglobulin G; IgA, immunoglobulin A. ^a^, ^b^, in the same row, values with the same- or no-letter superscripts mean no significant difference (*p* > 0.05), while values with different-letter superscripts mean significant difference (*p* < 0.05).

## Data Availability

Data will be made available on request.

## Abbreviations

ADFI	Average daily feed intake
ADG	Average daily gain
BTI	Bis-1,1-trifluoroacetoxy-iodobenzene
CD	Crypt depth
CGM	Corn gluten meal
FCR	Feed conversion rate
GCFF	Glutamine-enriched corn gluten meal fermented feed
Gln	Glutamine
OTU	Operational taxonomic units
PCoA	Principal coordinate component analysis
SBM	Soybean meal
UFF	Corn gluten meal unfermented feed
VCR	Villus height to crypt depth
VH	Villus height

## Appendix A

**Table A1:** Partial corn protein sequence information in Uniprot.

No.	Sequence	Position in Protein	Protein Name	Access No.
1	QPPRPQP	88–94	Glutein-2	P04706
2	LRQQCCQQL	151–159	Glutein-2	P04706
3	LQQQPQSGQVA	180–190	Glutein-2	P04706
4	AAQIAQQL	194–201	Glutein-2	P04706
5	LQQP	207–210	Glutein-2	P04706
6	LRQQCCQQQM	83–92	Zein-beta	P06673
7	AQQLQMMMQL	101–110	Zein-beta	P06673
8	ALMQQQQQLL	124–133	Zein-beta	P06673
9	LQQA	58–61	Zein-alpha A20	P04703
10	LFQQS	73–77	Zein-alpha A20	P04703
11	AQQLQQL	92–98	Zein-alpha A20	P04703
12	QQQQFL	116–121	Zein-alpha A20	P04703
13	LQQQL	135–139	Zein-alpha A20	P04703
14	QQQQLL	151–156	Zein-alpha A20	P04703
15	LQQQIL	170–175	Zein-alpha A20	P04703
16	LQLQQL	210–215	Zein-alpha A20	P04703

Note: Q, glutamine; P, proline; R, arginine; C, cysteine; L, lysine; S, serine; G, glycine; V, valine; A, alanine; I, isoleucine; M, methionine; F, phenylalanine.
